# Methodology for Anti-Gene Anti-IGF-I Therapy of Malignant Tumours

**DOI:** 10.1155/2012/721873

**Published:** 2012-02-14

**Authors:** Jerzy Trojan, Yuexin X. Pan, Ming X. Wei, Adama Ly, Alexander Shevelev, Maciej Bierwagen, Marie-Yvonne Ardourel, Ladislas A. Trojan, Alvaro Alvarez, Christian Andres, Maria C. Noguera, Ignacio Briceno, Beatriz H. Aristizabal, Heliodor Kasprzak, Huynh T. Duc, Donald D. Anthony

**Affiliations:** ^1^INSERM U542 and U602, Paul Brousse Hospital, Paris XI University, 16 Avenue. PV Couturier, 94807 Villejuif, France; ^2^Laboratory of Gene Therapy, Faculty of Medicine, Cartagena's University, Cartagena de Indias, Colombia; ^3^Faculty of Medicine, La Sabana University, Chia, Autopista Norte de Bogota, Colombia; ^4^Department of General Medical Sciences, Case Western Reserve University, 2085 Adelbert Road, Cleveland, OH 44106, USA; ^5^Cellvax, Veterinary National School, 7 Avenue General De Gaule, 94704 Maisons Alfort, France; ^6^Laboratory of Cell Engineering, Cardiology Institute, Moscow University, Cherepkowskaya Street, Moscow 12 1552, Russia; ^7^Department of Gene Therapy and Department of Neurosurgery, Collegium Medicum, Nicolas Copernic University, M. Curie Sklodowska Street, 85067 Bydgoszcz, Poland; ^8^Laboratory of Neurobiology, Faculty of Science, Orleans' University, 45067 Orleans, France; ^9^INSERM U930, Bretonneau Hospital, Tours' University, 2 Bd Tonnelle, 37044 Tours, France; ^10^Laboratory of Molecular Diagnostic, Pablo Tobon Uribe Hospital, UPB University, Medellín, Colombia

## Abstract

The aim of this study was to establish the criteria for methodology of cellular “anti-IGF-I” therapy of malignant tumours and particularly for glioblastoma multiforme. The treatment of primary glioblastoma patients using surgery, radiotherapy, and chemotherapy was followed by subcutaneous injection of autologous cancer cells transfected by IGF-I antisense/triple helix expression vectors. The prepared cell “vaccines” should it be in the case of glioblastomas or other tumours, have shown a change of phenotype, the absence of IGF-I protein, and expression of MHC-I and B7. The peripheral blood lymphocytes, PBL cells, removed after each of two successive vaccinations, have demonstrated for all the types of tumour tested an increasing level of CD8^+^ and CD8^+^28^+^ molecules and a switch from CD8^+^11b^+^ to CD8^+^11. All cancer patients were supervised for up to 19 months, the period corresponding to minimum survival of glioblastoma patients. The obtained results have permitted to specify the common criteria for “anti-IGF-I” strategy: characteristics sine qua non of injected “vaccines” (cloned cells IGF-I(−) and MHC-I(+)) and of PBL cells (CD8^+^ increased level).

## 1. Introduction

Current treatment options for patients with advanced malignant tumours, including brain tumour glioblastoma (mortality approaching 100%), such as surgery, radiation, or hormone therapy are limited in efficacy; therefore the search for new strategies: innovative chemotherapy [1], use of inhibitors, including antibodies, antisense oligonucleotides, short peptides, and other small molecules [2–4], or cellular immune therapy [5] constitutes a permanent challenge. 

**Figure 1:**
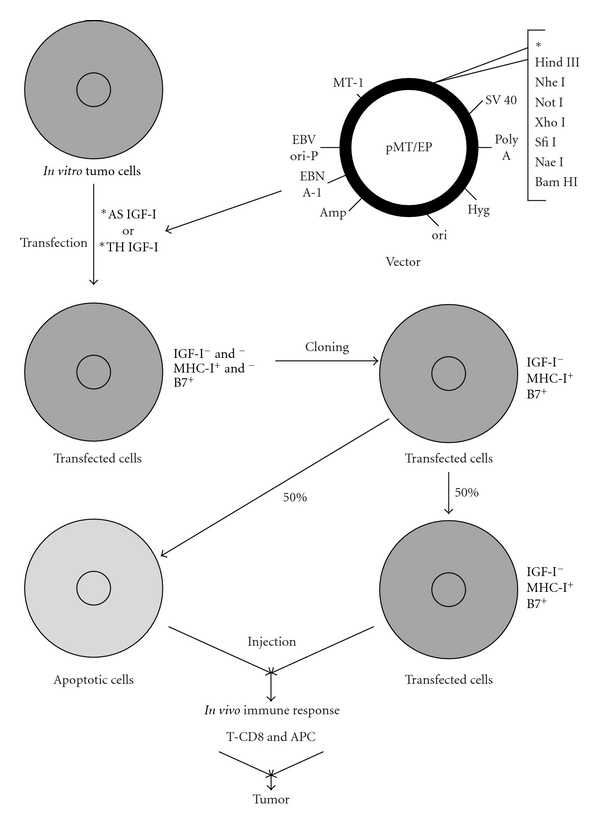
Mechanism of anti-gene anti-IGF-I (antisense/triple helix) therapy of malignant tumours. The case of glioblastoma therapy: hypothetically, the same mechanism should exist in the treatment of other tumours expressing IGF-I. The mechanism of antisense therapy is a combination of an augmentation of the immune antitumour response and of an inhibition of signal transduction pathway that is involved in the transformed phenotype of the tumour. Tumour cells are transfected *in vitro* with a vector encoding IGF-I cDNA in antisense orientation, or with a vector inducing a formation of triple helix IGF-I structure. The transfected tumor glial cells, in absence of IGF-I, become immunogenic-expressing MHC-I and B7 molecules, and apoptotic as follows. The expression of MHC-I is due to the presence of TAP1; the expression of B7 is related directly with signal transduction pathway: TK/IRS/PI3K/PKC; the phenomenon of apoptosis is also related with signal transduction pathway: TK/IRS/PI3K/AKT/Bcl2 [4, 13–18]. After *in vivo* injection, together with the antigen presenting cells, APC, they activate the T CD8 (CD8CD28) lymphocytes inducing immune antitumour response against the malignant glioma (expressing MHC-I) [4, 15, 19–22].

We have previously described the immune cellular/anti-gene anti-IGF-I approach [4], targeting IGF-I, the growth factor playing a principal role in the tumour growth processes [6]. Such strategy of anti-gene, of antisense or triple helix approach [7–9], has permitted to stop the development of the following animal tumours: glioma, hepatoma, melanoma and teratocarcinoma (containing three tissue derivatives) as well as to treat human gliomas, mediated by immune antitumour CD8***^+^*** T cells induced *in vivo *by injection of cellular “vaccines” presenting immunogenic characteristics (expression of MHC-I) (Figure 1) [4, 10–12].

The principal goal of this work—Phase I gene therapy of glioblastoma—was to establish the criteria of methodology for clinical trial based on principal results of previously described studies—the immune antitumour phenomenon observed in the antisense anti-IGF-I treatment of rat and human gliomas, and signaled by the increase of CTL CD8^+^ in the tumour tissue as well as in peripheral blood lymphocytes [4]. In the present work, we have used the strategy of combined antisense/triple helix technologies to prepare the anti-gene anti-IGF-I “vaccines” and investigate an immune response in treated patients with malignant tumours expressing IGF-I. Comparatively, the tumours representing three tissue derivatives were considered: principally neuroectodermal-glioblastoma, entodermal-liver and colon cancers, and mesodermal-cancers of ovary, uterus, and prostate [4, 23, 24].

## 2. Materials and Methods

### 2.1. Ethical Consideration

Human experiments were conducted in accordance with the Declaration of Helsinki (1964). The experiment was conducted with the understanding and the consent of the human subject. The responsible Ethical Committees have approved the experiments.

The approval for the gene therapy clinical trial (based on NIH clinical protocolno.1602, Bethesda, Maryland, 24. 11. 1993), containing scientific basis of methodology, cell therapy product standardization of preparation, detailed clinical protocol including inclusion criteria and exclusion criteria (i.e., HIV and EBV active infection), and the letter of agreement, was administrated by the Bioethical Commissions of the L. Rydygier Medical University, Bromberg (Bydgoszcz), Jagiellonian University, Cracow, Poland no. KB/176/2001, 28. 06. 2002, and (no. KBET/184/L/2000, 21. 09. 2000), La Sabana University, Chia, Colombia, no. P 004-10, 15. 12. 2010, Cartagena's University, Colombia, no. 3-19.10.2011, and registered by international Wiley Gene Therapy Clinical Trial database, Stockholm, no. 635 and 636 (J Gene Med, updated 2002). The protocol was verified by Ministry of Health, AFSSAPS Committee, Paris, France, 03. 06. 2005, and by NATO Science program 2003–2007 (no. LST 980517).

### 2.2. Preparation of Cell “Vaccines”

#### 2.2.1. Plasmids

The IGF-I antisense and triple helix technologies, both suppressing IGF-I expression, were used to construct episome-based plasmids either pMT-Anti-IGF-I expressing IGF-I RNA antisense, or pMT-AG inducing the IGF-I RNA-DNA triple helix, coming from pMT-EP “empty” vector [10, 25]. The cassette contains the Epstein-BarrVirus origin of replication and the gene encoding nuclear antigen I, which together drive extrachromosomal replication. In the pMT-AG triple helix, the cassette consists of a 23 bp DNA fragment cloned into the pMT-EP vector, which transcribes a third RNA strand forming a triple helix structure within the target region of the human IGF-I gene (Figure 1). The triple helix structure forming IGF-I RNA- DNA structure, giving rise to used IGF-I triple helix gene therapy approach, was largely described in previously published papers; moreover, the experimental data *in vitro* accompanied by control experiments constituted either by use of antisense technique or by use of control “empty” vectors were also performed [19, 25].

The vector and the cells transfected with these vectors were tested for the presence of DNA sequence of EBV virus—in the vector, the 4.4 Kb sequence of EBV is inserted. The tests of PCR EBV have given the negative results (Texcell-Institut Pasteur, ref. 114/01/1054D-02/07 and -01/03; report 27.03.1996). Although the testes were done in 1996, these results are still valuable because the total sequence of used vectors was never changed.

#### 2.2.2. Establishment of Primary Cell Cultures

The cancer cells were originated from surgically removed biopsies of primary malignant tumours as follows: glioblastoma (astrocytoma grade IV, glioblastoma multiforme), hepatocarcinoma (differentiated adenocarcinoma), colon carcinoma (differentiated adenocarcinoma), ovary carcinoma (cystadeno-carcinoma), uterus carcinoma (endometrial adenocarcinoma), and prostate carcinoma (adenocarcinoma, cytologic malignancy, grade III). Two cases of each malignant tumour were investigated. Surgical resections [10] were done in the University Hospital of Bromberg (Bydgoszcz), Poland. Primary cell lines originated from every biopsy were established during 3-4 weeks [19], simultaneously in three countries (Bromberg and Cracow, Poland, Paris, France, and Cartagena, Colombia).

 The removed cancer tissue material was vial to establish the cell culture if the biopsy was used before 24 hours following surgery. Cells were cultured in DMEM (GIBCO-BRL) supplemented with 10% FCS, 2 mM glutamine, 100 U/mL penicillin, and 100 ug/mL streptomycin, at 37°C and 5% CO_2_. In the case of glioblastoma and colon cancer, primary human cell lines established previously (CWRU, Cleveland, and Paul Brousse Hospital, Villejuif) have played a role of “cell line controls” for verifications of IGF-I presence (immunocytochemical reaction for IGF-I, using antibodies anti-IGF-I, and confirmed by RT-PCR), and MHC-I and B7 antigens absence (immunocytochemical or flow cytometry analysis using antibodies anti-MHC-I and anti-B7) [19, 26, 27].

RT-PCR (reverse transcriptase-polymerase chain reaction) technique was applied as described earlier [27]. RNA from cells was isolated using High Pure RNA Isolation Kit (Roche Diagnostics GmbH no.1828665). The applied components of RT PCR were used according Reverse Transcription System Promega Corporation (no. A3500). The following primers were used for RT PCR study of human IGF-I: 

forward primer IGF-I: GCATCTCTTCTACCTGGCGCTG, and reverse primer IGF-I: CAGGCTTGAGGGGTGCGCAATA (sequence according to “rgd” Human Genome Database).

We notice that the efficiency rate in the *in vitro* establishment of tumour cell lines was 100% [19, 27] and that this issue has not represented a limit in the number of patients that could be enrolled in this study. In addition, the quality control of the tumour cultured cells has concerned the test for mycoplasma, endotoxin, and aerobic and anaerobic bacteria.

#### 2.2.3. Transfection of Cell Lines

**Figure 2:**
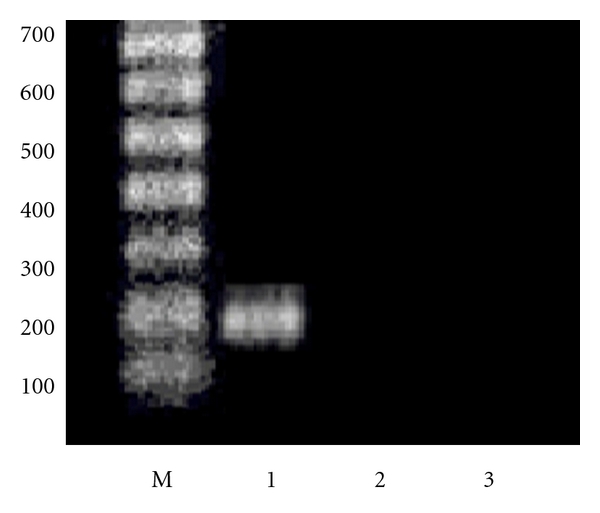
Expression of IGF-I in primary human glioma cell line. RT PCR technique. M—marker. 1—presence of IGF-I in parental non transfected cells (200 bp band of amplified DNA using IGF-I primer; see Methods). 2—absence of IGF-I expression in cells transfected with antisense anti-IGF-I vector. 3—absence of IGF-I expression in cells transfected with triple helix anti-IGF-I vector.

**Figure 3:**
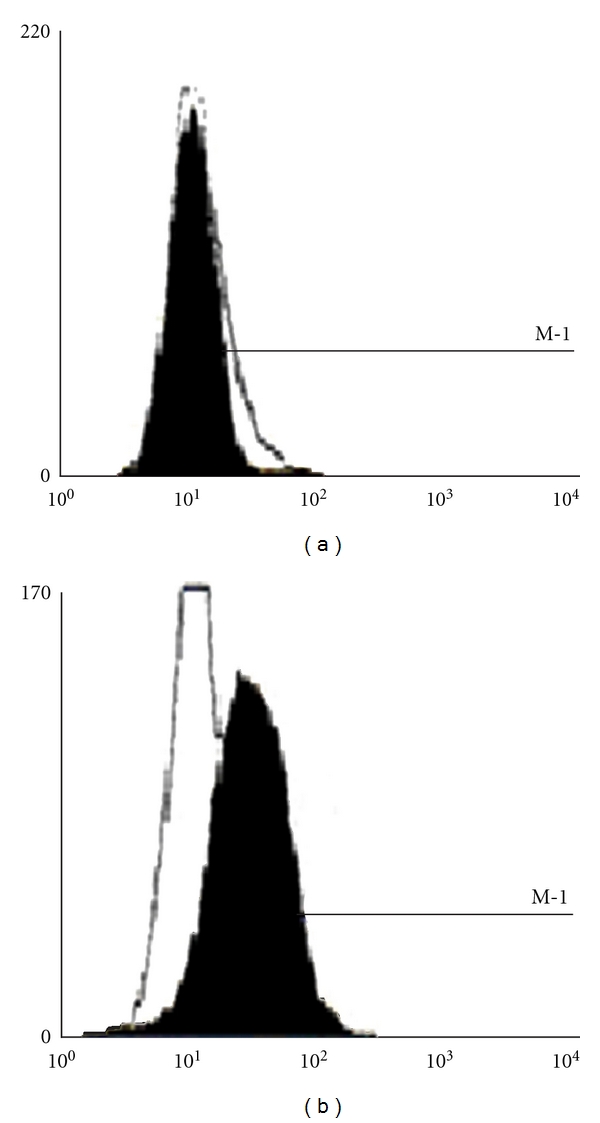
Expression of MHC-I in an established primary human glioma cell line. Flow cytometry analysis (FACScan Becton Dickinson). Panel left: parental nontransfected cells. Panel right: cells transfected with both antisense and triple helix anti-IGF-I vectors.

Using both antisense and triple helix anti-IGF-I expressing vectors, transfection was done during 2-3 weeks, by either Ca^++^/Ph technique or FuGENE 6 Transfection Reagent (Boehringer Mannheim) [19]. 48 hours after transfection, the selection of transfected cells was done in the presence of Hygromycin B (Boehringer Mannheim) at a concentration of 0,005 mg/mL. After one week, concentration of hygromycin B was changed to 0,015 mg/mL and progressively increased up to 0,15 mg/mL and maintained with each change of fresh medium over the next 2-3 months. Two weeks after transfection, cell lines derived of the same tumour were verified for absence of IGF-I using immunocytochemistry technique, confirmed by RT PCR technique (Figure 2), and for presence of MHC-I and B7 molecules using flow cytometry analysis (Figure 3): monoclonal antibodies, labeling human MHC-I (HLA), MHC-II, CD80, and CD86 (B7) antigens were used for direct immunostaining (Becton Dickinson Pharmingen) [19, 27]. The expression of IGF-I, MHC-I, and B7 in nontransfected and transfected separately “antisense” and “triple helix” cancer cell lines (Figure 2) was verified in the laboratories of Bromberg and Krakow (Poland), Cleveland (USA), Villejuif (France), and Medellin and Bogota (Colombia). The cell lines transfected with “empty” vector have constituted a negative control for both “antisense” and “triple helix” lines.

#### 2.2.4. Cloning of Cells

The clones—transfected “antisense” and “triple helix” cells—expressing MHC-I and B7 molecules were used for preparation of cell vaccines (in general, 40–50% of clones were positively stained for MHC-I, and 50–60% of clones were negative for MHC-I [11]). The cultures of these clones, four weeks after transfection, have presented about 50–60% of apoptotic cells, and 40–50% of nonapoptotic cells which were IGF-I(−), MHC(+), and B7(+) (Figure 1) [11, 19]. The apoptosis was verified as described earlier [11, 28].

The established cell cultures were divided in three parts. The first part, 200 000 cells, was used for the preparation of cell membranes, prepared according to the technique of Matlib et al. [29]. These cell membranes have constituted the material for first, noncell, “membrane vaccination”. When the cells growing in culture were numerous enough, 3–5 million “antisense cells” and “triple helix cells”, respectively, one part of them was used for “cellular vaccination”—injection of 3 million of cells: 1.5 million of “antisense cells” mixed with 1.5 million “triple helix cells”, and another part was frozen as “back-up” in liquid nitrogen.

### 2.3. Vaccination of Cancer Patients

The principal clinical trial concerned glioblastoma patients. Six patients in University Hospital of Bydgoszcz and six patients in CWRU Hospital of Cleveland were treated, respectively. The patients in Bydgoszcz were divided in three groups of two cases each (considering that this clinical trial was the beginning of Phase 1 presenting a limited number of patients, the statistical analysis was not included in the study). In every group the age of patients was about twenty years and sixty years, respectively (the individual characteristics are not a subject of the presented work). In the first group, the patients treated by surgery, radiotherapy, and chemotherapy (low dose chemotherapy-temozolomide 75 mg/m^2^/day, applied during the period of radiotherapy) have followed three successive subcutaneous injections of cellular membranes isolated from IGF-I antisense/triple helix-transfected cells (membrane vaccinations). In the second group after surgery, radiotherapy, and chemotherapy (low dose), the first membrane injection was followed by two successive subcutaneous “cellular vaccinations” composed of IGF-I antisense/triple helix-transfected cells—containing both apoptotic and nonapoptotic cells. The third group, a control group, was treated by surgery, radiotherapy, and chemotherapy. On the other side, six glioblastoma patients in Cleveland were treated by unique therapy: surgery, radiotherapy, and chemotherapy were followed by successive subcutaneous “cellular vaccinations” composed of IGF-I antisense-transfected cells containing both apoptotic and non apoptotic cells. In parallel, the patients with cancers of liver, colon, uterus, ovary, and prostate (Bydgoszcz) have followed the treatment composed of surgery, radiotherapy, and successive “cellular vaccinations” (see Section 3.3).

The injections were done with interval of four-five weeks. 48 hours before every vaccination, the membrane or cell pellets (“membrane” or “cellular” therapies) were irradiated with 5000 cGy gamma (Co60 or Cs137) [10]. The membranes or cells were injected subcutaneously, into the left arm of operated cancer patients, in 1 mL of PBS solution. The blood was collected three times: before vaccinations and after every of two cell vaccinations (2-3 weeks after the second and the third injection).

Flow cytometric analysis of PBL cells was done as follows: peripheral blood lymphocyte (PBL) typing was performed after hemolysis by incubation of peripheral blood with monoclonal antibodies against the cell antigens: CD3, CD4, CD5, CD8, CD8^+^11b^+^, CD8^+^11b^−^, CD8^+^28^+^, CD19, CD3^−^(16 + 56) + (NK), CD25, CD44, and CD45 (Becton Dickinson Pharmingen, direct immunostaining). Paraformaldehyde-fixed cells were examined in FACSscan BD cytometer. Double direct immunotyping with pairs of monoclonal antibodies conjugated with FITC and PE were used. Lymphocyte gate was defined according to the CD45 back gating. Data were presented as percentage of positive cells [4, 19]. The PBL labelling of cells was done simultaneously in two laboratories (Bydgoszcz and Villejuif).

## 3. Results

### 3.1. Preparation of Cell “Vaccines”

**Figure 4:**
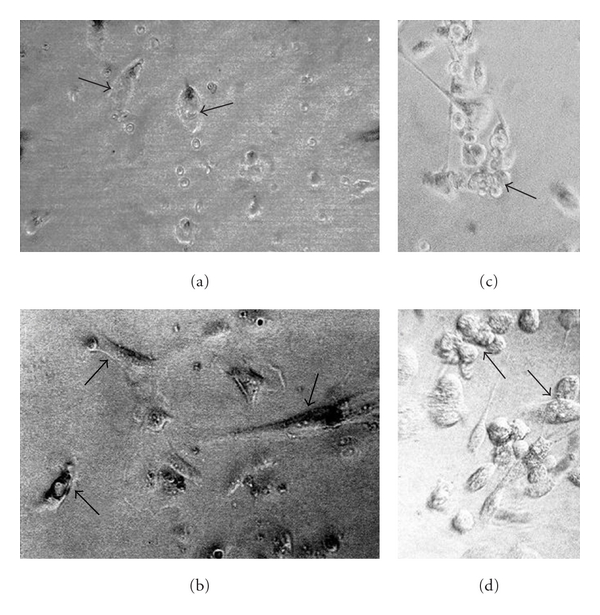
Examples of *in vitro* culture of primary human cancer cells derived from surgical biopsies. (a) and (c) Primary ovary cystadenocarcinoma-derived cells. (b) and (d) Primary prostate adenocarcinoma-derived cells. Parental ovary cancer cells (a) and prostate cancer cells (b) are attached efficiently in the fourth day of culture (arrows). “Antisense/triple helix” anti-IGF-I ovary cancer cells (c) and prostate cancer cells (d), both twenty days after transfection, form the established lines characterized often by the clusters of round apoptotic cells, becoming progressively small (c, arrow; d, arrow up). They are accompanied by nonapoptotic and more voluminous cells (d, arrow down) presenting generally elongated shape (c and d). The transfected cells are always different from nontransfected parental cells (a, b), as it was demonstrated previously in cases of human glioma and hepatoma cell lines established from primary tumours of glioblastoma and hepatocarcinoma [30, 31] X400.

The primary human cell lines derived from all types of cancer biopsies—primary tumours—were successfully established following the technique described earlier concerning glioblastoma, hepatoma, and colon cancer [4, 19, 26]. Each cell line was subcloned to obtain IGF-I positive clones (the percent of IGF-I positive cells in the human cell lines ranged from 50 to 70%). All the primary cell lines were also transfected, showing a change of phenotype. The examples of primary cell lines as well as of transfected cell lines of ovary and prostate cancers are shown in Figure 4.

**Figure 5:**
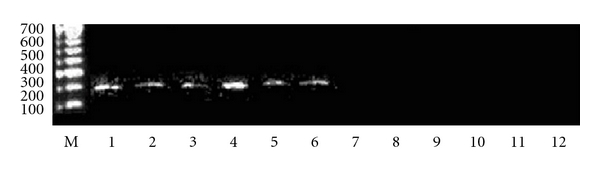
Expression of IGF-I in human cancer cell lines. RT PCR technique. M—marker. Lines 1 to 6—presence of IGF-I in parental nontransfected cancer cell lines derived from 1—glioblastoma, 2—hepatocarcinoma, 3—colon adenocarcinoma, 4—ovary cyst adenocarcinoma, 5—uterus endometrial adenocarcinoma, and 6—prostate adenocarcinoma (200 bp band of amplified DNA using IGF-I primer; see Section 2. Lines 7 to 12—absence of IGF-I expression in cancer cells transfected with both antisense and triple helix anti-IGF-I vectors: 7—glioblastoma, 8—hepatocarcinoma, 9—colon adenocarcinoma, 10—ovary cyst adenocarcinoma, 11—uterus endometrial adenocarcinoma, and 12—prostate adenocarcinoma.

IGF-I expression was verified before and after transfection of all types of cancer cells: using RT PCR technique, analysis of RNA of cancer cells four weeks after transfection was compared to RNA of parental cells (Figure 5). Then the transfected cells (not expressing IGF-I), serving as “vaccines” for different types of cancers, were cloned to obtain MHC-I positive cell lines as previously described in the case of human glioblastoma-transfected cells [19]. The transfected MHC-I positive cancer cells have also expressed B7 antigen with exception of hepatoma-transfected cells as described earlier [11]. Human cancer cells transfected with “empty” vector, pMT/EP, serving as negative control, were stained positively for IGF-I and negatively for MHC-I and B7.

### 3.2. PBL Typing in Treated Cancer Patients

**Figure 6:**
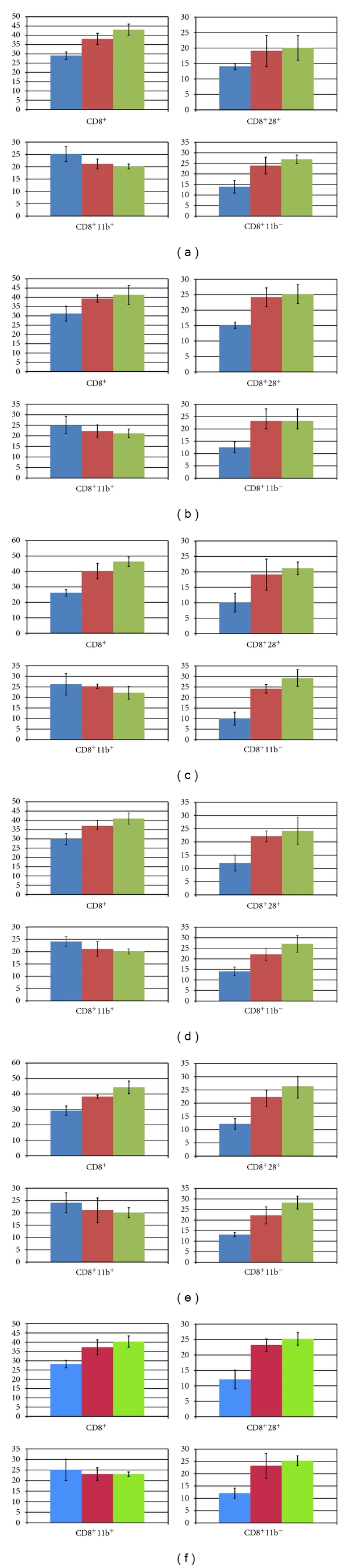
Flow cytometric “FACS” peripheral blood lymphocyte CD marker patterns following cellular gene therapy in human cancers. (a) glioblastoma multiforme; (b) hepatocarcinoma; (c) colon adenocarcinoma; (d) ovarian carcinoma; (e) uterine adenocarcinoma; (f) prostate adenocarcinoma. CD molecules were labelled in peripheral blood lymphocytes (PBLs) obtained from prevaccinated and “vaccinated” cancer patients. Each of the first column corresponds to data obtained before vaccinations; each second and third column corresponds to data obtained after one and two successive cellular vaccinations (IGF-I antisense/triple helix cells). Two cases of each of the designated cancers were examined (bar graphs represent the median value of the two cases). Data are expressed as percent of positive cells when compared to the isotype control. Difference in percentage of CD8^+^ CD11b^−^ and CD8^+^ CD28^+^ subpopulations before and after vaccination was strongly significant with a range of *P* from 0.001 to 0.02 according to the Student's *t*-test and weakly significant concerning the decreasing CD8^+^ CD11b^+^ subpopulation from the relevant patients. The *P* value for CD8^+^, CD8^+^28^+^, and CD8^+^11b^−^ (below 0.01) is illustrated in the bar graph for statistical significance. (The original FACS data concerning PBL cells are in the archives of Collegium Medicum of Nicolas Copernic University, Bromberg, Poland; FACS data (*n* = 504) corresponding to the labeling of CD3, CD4, CD5, CD8, CD8^+^11b^+^, CD8^+^11b^−^, CD8+28^+^, CD19, CD3^−^(16 + 56) + (NK), CD25, CD44, and CD45.)

Clearcut phenotypic changes in peripheral blood lymphocytes (PBLs) were observed in all cancer diseases treated with “cellular therapy”: after the first cell vaccination, the increase of CTL CD8^+^, particularly CD8^+^11b^−^, was observed. There was a characteristic switching from CD8^+^11b^+^ to CD8^+^11b^−^ which was practically not significant in the group of “membrane therapy” applied for two glioblastoma patients. This increasing switching was also observed after the second cell vaccination in all treated cancers. Moreover, the PBL cells have demonstrated, in all types of tumour diseases, an increasing expression of cell surface markers CD8^+^CD28^+^ confirming the effectiveness of “cellular therapy” (Figures 6(a), 6(b), 6(c), 6(d), 6(e), and 6(f)). The results concerning other studied CD molecules as CD3 or CD19 and CD45 (data not shown) were no significant in all treated cancers; in the case of CD4 slightly decreased values were registered (as only two cases of each tumour were studied, these observations not be statistically valuable). That is important to add that no significant change in CTL CD8 level was observed as well before and after the surgery and before and after radiotherapy treatments as compared to “cellular therapy”. (The control “empty” vector, without antisense anti-IGF-I cDNA, used to prepare “vaccines” in animal models previously described, has never shown influence for immune antitumour response [32, 33].)

### 3.3. Median Survival of Treated Glioblastoma Patients

The results obtained in the University Hospital of Bromberg were as follows: two glioblastoma patients included in the group of “cellular therapy” have survived 19 and 24 months, respectively (beginning from day 0—diagnosis of malignant glioma, followed by surgery, radiotherapy, and antisense/triple helix cell injections). Two glioblastoma patients included in the group of “membrane therapy” have survived 9.5 and 10 months, respectively, starting from surgery followed by radiotherapy and injection of cell membranes. The results observed in the group of glioblastoma patients treated with “membrane therapy” were not so different from those obtained in the third group treated by classical therapy; in the last group median survival was as 10 and 11 months. For this reason, admitting that the group of glioblastoma patients treated with antisense/triple helix cell injection has given the significant results, all other cancer patients (age 20–65 years: two cases of liver, colon, ovary, uterus, and prostate cancer diseases) were treated, after surgery and radiotherapy with this type of “cellular therapy”. Moreover the period of 19 months was chosen as the end of clinical observations in all treated cancer patients. At 19 months, all these cancer patients were alive and the treatments were well tolerated (we do not include the details of clinical observations concerning different types of treated cancers, because it is not the subject of this work). The only secondary observed effect including glioblastoma patients was that of increased temperature up to 38-39°C persisting during two-three days after every of cell vaccination.

 Among the patients treated in USA (University Hospitals of Cleveland) with anti-gene anti-IGF-I “cellular therapy” (two cell injections), two of the treated patients forming a group of maximum median OS have both survived 19 months. The other group of three patients have not responded so positively to the therapy, showing the median survival compared with that of “membrane” therapy. The therapy done in USA has shown that the number of cell vaccinations (between one and four) was not related to the median OS. Concerning serial MRI/CT performed in USA patients: 1-2-month intervals before vaccine showed continuous growth of the intracerebral tumour. MRI one month after vaccination showed first evidence of an unequivocal decrease in size of tumours viewed by radiology in University Hospitals of Cleveland. Moreover, all patients treated in USA had advanced disease with cerebral edema at the time of first treatment with vaccine and also were receiving treatment with high dose of decadron or related steroids to reduce the effect of CNS edema. This of course has caused further jeopardy to the immune system and can explain the relatively negative results in three last treated cases.

## 4. Discussion

New ways of treating malignant tumours, and very efficient chemotherapies in particular, are constantly being investigated; the best example is that of temozolomide introduced in glioblastoma treatment by Stupp et al. [1]. This type of chemotherapy has permitted to increase the median survival up to 15–18 months of glioblastoma patients, showing a real success comparing to current 8–11 months of median survival using surgery and radiotherapy only [1, 2, 4].

The gene therapy may represent a novel approach for cancer therapy. The elucidation of the molecular biology of cancer cells in recent years has progressively identified the different molecular pathways altered in various cancers. Activation of the PI3K/AKT/GWK3/GS pathway is mediated by some tyrosine kinase receptors, under the control of several growth factors and cytokines as EGF, PDGF, VEGF, TGFbeta, CSF, and especially IGF-I, whose receptor, IGF-I-R, plays a principal role in the tumour growth process [4, 6, 34]. For most of the pathways that have been disclosed, it has been a problem to develop selective molecules having a relevant clinical impact in malignant diseases, including uncured glioblastoma [35]. To target specific genetic defects, the antisense oligonucleotides have become one of the important anticancer approaches used in clinical trials [36].

The glioblastoma, as well as other malignant tumours, was recently successfully treated by antisense therapy targeting TGF beta, using either antisense anti-TGF beta expressing vector [37] or particularly applying the oligodeoxynucleotides [38, 39]. Using phosphorothioate TFG beta 2 antisense oligonucleotides (AP-12009), an international phase II/III study was initiated in patients with TGF beta-overexpressing tumours such as high-grade gliomas, and by 2005-2006, the trial was ongoing in over 140 patients with anaplastic astrocytoma (AA) or glioblastoma; the treatment was very well tolerated. In 2007, at that time overall survival was 24 months, and in the control group, survival was 20 months [38, 40]. Results from the clinical trials concerning other tumours overexpressing TGF beta were also recently published (pancreatic carcinoma, metastatic melanoma, or advanced colorectal carcinoma); the treatment was well tolerated in all types of tumour diseases [38]. Other antisense approaches of malignant tumour treatment have been developed recently, since 2001, especially those of antisense anti-IGF-I-Receptor [6, 41]. AS IGF-I-R strategy of treatment of glioblastoma [41] was not continued. It seems that this therapy could be more efficient if the cell “vaccines” were prepared after cloning of IGF-I-R antisense cells for MHC-I expression.

In anti-gene anti-IGF-I approach, we have applied both antisense and triple helix technologies, permitting to stop simultaneously the expression of IGF-I on translation and transcription levels [4]. Moreover, *in vivo *AS IGF-I approach was also developed [42]; 45 patients with PHC were cotransfected *in vivo *with antisense IGF-I expression vector and sense B7.1 expression vector. At two years following treatment of PHC stage II, there was marked reduction in tumour recurrence—from 62 to 20%.

In antisense anti TGF-beta technique as well as in anti-IGF-I and IGF-I-R approaches, the immune antitumour response was signalled as a principal mechanism of antisense technology inhibiting growth factors and their signalling pathway [19, 37]. The mechanism of antisense therapy targeting growth factors and their receptors is a combination of an augmentation of the immune antitumour response and of an inhibition of the signal transduction pathway—PI3K/AKT/GWK3/GS—that is involved in the transformed phenotype of the tumour [4, 13]. As far as PI3K/AKT/GWK3/GS pathway (in relation with glioma) is considered, it was recently demonstrated that in experimental antisense antiglycogen synthetase, GS, tumour therapy, the transfected AS GS cells were also immunogenic (MHC-I expression) [13, 14]. Anyway in AS GS strategy an immune antitumour response was not as striking as when using AS IGF-I approach. This shows that AS IGF-I appears as a dominant tool for the arrest of tumour progression. Moreover, targeting IGF-I instead of IGF-I receptor seems more efficient: because of downstream elements involved in the IGF-I-R transduction pathway, signals from IGF-I-R can be inappropriate or exaggerated [34]. Nevertheless, if crosstalk of IGF-I's related different pathways is considered, IGF-I, through its binding to IGF-I-R, which activates PI3K/AKT transduction cascade, has been reported to block the apoptosis pathway (IRS/PI3K/AKT/Bcl or AKT/GSK3 or Ca^2+^ or caspases) [4, 6]. The final result of AS IGF-I approach including the TK/PI3K/AKT pathway elements inhibition is an immune response mediated *in vivo *by lymphocytes T CD8 and APC cells [4]. As to PI3K/AKT/GWK3/GS pathways of IGF-I AS or TGFbeta AS therapies, we cannot avoid the relation with PI3K/PKC/RAF/MAPK chain, and we cannot exclude that the inhibition of TK/PI3K/AKT pathway using AS IGF-I approach can be reinforced by “side” effect of MAPK inhibition [4]. The inhibitors of RAF targeting the ATP binding site, as well as the inhibitors of MAPK at a non-ATP site, were also introduced in cancer clinical trials [32, 43].

Using described here IGF-I antisense/triple helix strategy, all treated patients have well tolerated the three injections of transfected cancer cells. The PBL cells have shown an increase in CD8^+^CD28^+^ molecules with a characteristic switching from CD8^+^11b^+^ to CD8^+^11b^−^ phenotype, observed after two cell vaccinations, reflecting the enhanced activation of cytotoxic T-cells in blood. These results concerning the switching CD8^+^11b^+^ to CD8^+^11b^−^ in different treated tumours have confirmed previously obtained data in glioblastoma and hepatoma treatment using antisens anti-IGF-I approach [4, 28]. The work in progress has also shown, in different treated tumours described here, an increased percentage of T CD25 (interleukin-2 receptor), in the context of CD4, which has confirmed the results obtained in glioblastoma treatment [4]. (Currently we are engaged in Phase II with 60 patients treated for different types of cancers mentioned earlier, including lung and stomach cancers.) The only secondary observed effect was increased temperature, 38-39°C, confirming the immune response induced by antisense/triple helix “vaccines”. The specificity of immune response was every time confirmed as follows. As demanded by approved clinical trials (see higher mentioned Bioethical Committees from 1993 to 2010), the removed PBLs of patients were tested *in vitro* for immune response after contact with autologous tumor cells to demonstrate antitumor activation throught high percentage of specific T cells observed after vaccination compared to controls—before vaccination (the tumoral cells were labeled with Cr51 before the test of lyses in the presence of CTL cells [33, 44]; data not shown).

Regarding injection of cell membranes, the switching mentioned earlier was not significant. The challenge of injection of membranes, isolated from IGF-I antisense/triple helix-transfected cells expressing MHC-I, has proved that the whole transfected cell population is necessary to produce an *in vivo* antitumour effect. At first, the cytoplasm of the transfected cells contains the IGF-I antisense RNA and IGF-I triple helix RNA-DNA structures constituting the principle of anti-gene cellular therapy [25, 45]. Next, the cellular therapy described here has shown that both cell populations, as well MHC-I and B7 expressing transfected cells as apoptotic cells, are necessary to induce *in vivo* an immune antitumour response involving APC activating CD8^+^ T cells [4, 11, 30, 45, 46]. (It was previously demonstrated that doubly transfected cells, using antisense anti-MHC-I and anti-B7 vectors, lose their apoptotic and immune antitumour characters [19]. It was, this way, shown that both processes, immunogenicity (MHC-I and B7 expression) and apoptosis, “work” together [11, 19, 31].) On the other side we have previously compared the efficiency of gene therapy—using the injection of IGF-I AS nucleotides, and that of described here “cellular therapy” much more promising. In gene therapy approach, after the injection of IGF-I AS nucleotides directly to the tumor, the cancer cells internalizing AS nucleotides could not become immediately immunogenic to induce the rapid immune response, and for the same reason to develop the efficient apoptosis. Moreover, in the gene therapy, the cotransfection with B7 expression vector was necessary to reinforce the immune response [28, 42].

As far as the relationship between anti-gene anti-IGF-I technology and immunogenicity is considered, the absence of IGF-I synthesis in “antisense-” and “triple-helix”-transfected cells could lead to a compensative increase in IGF-I receptor (tyrosine kinase); IGF-I and IGF-II present in foetal calf serum of culture medium, as well as intracellular IGF-II can interact with the type I receptor [47]. Indeed, the increase of IGF-I receptor level could explain the expression of B7. There is a known relation between the signal transduction pathway of tyrosine kinase and the induction of B7 molecules: enhancement in B-7 costimulation through a cAMP mechanism linked to tyrosine kinase of the CD 28 receptor has been previously reported [15]. (The costimulatory B7 molecule in antigen presenting cells (APCs) is bound to the counter-receptor CD28 and/or CTLA4 expressed on the T-cells [20, 48, 49].) B7 was present in different antisense and triple helix anti-IGF-I transfected cancer cells but absent in transfected human hepatoma and in previously described murine hepatoma [47]. Those results have also demonstrated that the expression of MHC-I in human-transfected hepatoma cells was much higher than that in transfected human glioma cells. This strong expression of MHC-I in human-transfected hepatoma lines (5 times, compared to human-transfected glioma lines) could explain that the presence of MHC-I was sufficient to induce T CD8 lymphocytes response in the absence of B7 antigen [28]. To summarize the immune antitumour mechanism of anti-gene anti-IGF-I strategy, this aspect was published previously (i.e., [4, 28]). As far as largely studied glioma treatment is considered, and similarly other concerned tumours, the mechanism concerns the reaction between activated lymphocytes expressing CD8CD28 and immune molecules MHC-I and B7. The following chain reaction could occur: cultured cloned glioma cells (IGF-I(+), MHC-I(−), B7(−)) *⇒* cultured transfected anti-gene IGF-I cells (IGF-I(−), MHC-I(+), B7(+)) *⇒* injection (glioblastoma patients) *⇒* induction of CTL CD8(+) CD28(+) *⇒* destruction of injected transfected anti-gene IGF-I cells (IGF-I(−), MHC-I(+), B7(+)), and arrest of a solid glioma tumour (IGF-I(+/−), MHC-I(+), B7(+)) (see also caption of Figure 1).

The immune criteria of used vaccines are strongly related to the preparation of cancer cells to be used as vaccine. Cancer cells cultivated under stem cell-permissive conditions more closely reflect the tumor of origin, including the genetic profile, than the parental tumor adherently growing cells under conventional cultivation conditions [50, 51]. In our experimental clinical trial, to avoid this effect of “contamination” increased by numerous passages, the primary cancers cells and transfected cancer cells were systematically cloned after every passage to obtain *in vitro*, in the first case 100% IGF-I(+), MHC-I(−), and in the second case 100% IGF-I(−), MHC-I(+) expression. The vaccines prepared with no cloned cells (both cancer cells and transfected cancer cells) did not induce the immune response* in vivo* in animals and in patients. 

Moreover, to produce their immune antitumour effect, the vaccines were composed of—criteria sine qua non— the mixture of higher characterized cancer-transfected cells and of cancer cell-derived apoptotic cells.

We need to add that if the all clinical results concerning this work could be cited, we would be obliged to give the summary of clinical data concerning 42 cancer patients and their laboratory data analyzed in this paper (Bromberg and Cleveland), including corresponding original FACS of PBL cells labeling of every patient. The article concerning only clinical results of every patient (clinical data of treated cancer patients are in archives of four hospitals, in Bromberg, Cracow, Cleveland, New York, Shanghai, and Bangkok; 70 patients only in Bromberg) including detailed inclusion and exclusion criteria, clinical and laboratory data*— *PCR and RT PCR of IGF-I; immunocytochemistry of cytokines, growth factors, and MHC-I and B7 molecules; and blood test of every patient—, will be published as separate article with obligatory statistics, not treated in the presented manuscript.

## 5. Conclusions

Our presented work concerns the criteria established for methodology of anti-IGF-I gene therapy analyzing our different previous basic and clinical results obtained in Europe, USA, and Asia, following our NATO science program (see Acknowledgment), and published recently [4, 27, 28], permitting to start Phase I and II in South America (Colombia) and Africa (Senegal). This way we have established the common criteria for selection of vaccines (expression of IGF-I, MHC-I, B7) and of PBL cells markers (CD8^+^-related molecules) in patients presenting the arrest of growing tumours. The various therapies in the treatment of cancer are still experimental [52]. A number of strategies for inhibiting gene expression have been developed including the triple helix approach, antisense cDNA, and oligodeoxynucleotides. Among the new strategies in the efforts of treating malignant tumours expressing different growth factors, and more specifically IGF-I, TGF beta, VEGF, or EGF [3, 6, 35, 37], the anti-gene therapy approach, either antisense or triple helix, appears as a promising solution [39]. Although in the presented work only limited numbers of glioblastoma patients were treated, the clinical results obtained are positive (minimum survival has reached 19 months). The anti-gene anti-IGF-I therapy, giving a strong immune antitumour response in different comparatively studied tumour diseases, presents all characteristics of cell immunotherapy (CD8^+^and CD28^+^ expression in T lymphocytes, and MHC-I and B7 expression in “vaccine” cells) including apoptotic phenomenon [4, 15, 45, 53]. We suggest that anti-gene cell therapy, giving comparable results to those of currently applied chemotherapy, inhibitors, or antibodies [1, 2, 4, 54], could be used either alone [39, 55] or as combined therapies, that is, antisenses targeting simultaneously different elements of growth factors signalling pathway [13, 14, 32, 43], or as antisense/chemotherapy. The combined anticancer strategies considering the role of immune antitumour response [35, 56–62], including study of control CD8(+) T-cell effector functions [63], new tools of cell transfection [64] and especially the search for new oncoproteins [65], and growth factor targets [6, 14, 35, 66, 67], appear as the near future challenge. Among growth factors, targeting IGF-I system in relation with cancer therapy constitutes a permanent basic and clinical research [68–70].
