# Erratum to “Methodology for Anti-Gene Anti-IGF-I Therapy of Malignant Tumours”

**DOI:** 10.1155/2012/765703

**Published:** 2012-08-13

**Authors:** Jerzy Trojan, Yuexin X. Pan, Ming X. Wei, Adama Ly, Alexander Shevelev, Maciej Bierwagen, Marie-Yvonne Ardourel, Ladislas A. Trojan, Alvaro Alvarez, Christian Andres, Maria C. Noguera, Ignacio Briceno, Beatriz H. Aristizabal, Heliodor Kasprzak, Huynh T. Duc, Donald D. Anthony

**Affiliations:** ^1^INSERM U542 and U602, Paul Brousse Hospital, Paris XI University, 16 Avenue PV Couturier, 94807 Villejuif, France; ^2^Laboratory of Gene Therapy, Faculty of Medicine, Cartagena's University, Cartagena de Indias, Colombia; ^3^Faculty of Medicine, La Sabana University, Chia, Autopista Norte de, Bogota, Colombia; ^4^Department of General Medical Sciences, Case Western Reserve University, 2085 Adelbert Road, Cleveland, OH 44106, USA; ^5^Cellvax, Veterinary National School, 7 Avenue General De Gaule, 94704 Maisons Alfort, France; ^6^Laboratory of Cell Engineering, Cardiology Institute, Moscow University, Cherepkowskaya Street, Moscow 12 1552, Russia; ^7^Department of Gene Therapy and Department of Neurosurgery Collegium Medicum, Nicolas Copernic University, M. Curie Sklodowska Street, 85067 Bydgoszcz, Poland; ^8^Laboratory of Neurobiology, Faculty of Science, Orleans' University, 45067 Orleans, France; ^9^INSERM U930, Bretonneau Hospital, Tours' University, 2 Bd Tonnelle, 37044 Tours, France; ^10^Laboratory of Molecular Diagnostic, Pablo Tobon Uribe Hospital, UPB University, Medellín, Colombia

The authors would like to make the following corrections.

In page 2, paragraph 2, Materials and Methods, first column, 34th line, it should be *Cartagena's University Hospital del Caribe (preclinical study)* instead of Cartagena's University, second column, 29th line, it should be *Bogota, *instead of Cartagena.

